# Characterization of new cristamonad species from kalotermitid termites including a novel genus, *Runanympha*

**DOI:** 10.1038/s41598-021-86645-w

**Published:** 2021-03-31

**Authors:** Racquel A. Singh, Vittorio Boscaro, Erick R. James, Anna Karnkowska, Martin Kolisko, Gregory S. Gavelis, Noriko Okamoto, Javier del Campo, Rebecca Fiorito, Elisabeth Hehenberger, Nicholas A. T. Irwin, Varsha Mathur, Rudolf H. Scheffrahn, Patrick J. Keeling

**Affiliations:** 1 Department of Botany, University of British Columbia, Vancouver, BC Canada; 2 Institute of Evolutionary Biology, Faculty of Biology, Biological and Chemical Research Centre, University of Warsaw, Warsaw, Poland; 3 Institute of Parasitology, Biology Centre, Czech Academy of Sciences, České Budějovice, Czech Republic; 4 Department of Biochemistry, Purdue University, West Lafayette, IN USA; 5 Institut de Biologia Evolutiva (CSIC-Universitat Pompeu Fabra), Barcelona, Catalonia Spain; 6 Ocean EcoSystems Biology Unit, RD3, GEOMAR Helmholtz Centre for Ocean Research, Kiel, Germany; 7 Fort Lauderdale Research & Education Center, University of Florida, Davie, FL USA

**Keywords:** Taxonomy, Symbiosis

## Abstract

Cristamonadea is a large class of parabasalian protists that reside in the hindguts of wood-feeding insects, where they play an essential role in the digestion of lignocellulose. This group of symbionts boasts an impressive array of complex morphological characteristics, many of which have evolved multiple times independently. However, their diversity is understudied and molecular data remain scarce. Here we describe seven new species of cristamonad symbionts from *Comatermes*, *Calcaritermes*, and *Rugitermes* termites from Peru and Ecuador. To classify these new species, we examined cells by light and scanning electron microscopy, sequenced the symbiont small subunit ribosomal RNA (rRNA) genes, and carried out barcoding of the mitochondrial large subunit rRNA gene of the hosts to confirm host identification. Based on these data, five of the symbionts characterized here represent new species within described genera*: Devescovina sapara* n. sp., *Devescovina aymara* n. sp., *Macrotrichomonas ashaninka* n. sp., *Macrotrichomonas secoya* n. sp., and *Macrotrichomonas yanesha* n. sp. Additionally, two symbionts with overall morphological characteristics similar to the poorly-studied and probably polyphyletic ‘joeniid’ Parabasalia are classified in a new genus *Runanympha* n. gen.: *Runanympha illapa* n. sp., and *Runanympha pacha* n. sp.

## Introduction

Parabasalians (Parabasalia) are a diverse group of microaerophilic flagellated protists with reduced mitochondria that are mainly obligate symbionts or parasites of animals^1^. Historically, parabasalians were divided into two classes: the hypermastigids, which contain large cells with great structural complexity and hundreds or thousands of flagella, and the trichomonads, which are relatively simple cells with 3–6 flagella per mastigont (a structural complex comprised of all the organelles associated with the flagella)^1,2^. However, molecular phylogenies show that these two classes are not monophyletic^3–5^. Consequently, species originally classified within the hypermastigids and trichomonads are now placed into one of six different recognized classes^1^.

The largest of these classes in terms of the number of genera and species described is the Cristamonadea, which encompasses over 30 described genera and over 150 known species^2,4,6^. In comparison, the next largest class, the Trichomonadea, is comprised of roughly 18 genera and 130 species^1^. The cristamonads are symbionts that live in the hindguts of wood-feeding termites, as part of a nutritional symbiosis in which they digest the lignocellulose that makes up the majority of the host diet^7–10^. Ancestrally, the cristamonad body plan was most likely a relatively simple karyomastigont system with four flagella associated with the nucleus, basal bodies, axostyle, parabasal bodies, and cresta, not unlike that of their closest relatives in the class Tritrichomonadea^4^. However, throughout cristamonad evolution either the entire karyomastigont system or parts of the system have multiplied in several different lineages in parallel, giving rise to large and complex forms that can be mononucleate or multinucleate, and may have single or multiple karyomastigont systems with 3–2000 flagella^4^.

This repeated parallel evolution of large and complex structural elements has allowed the cristamonads to become the most morphologically diverse of all the parabasalian lineages^11–13^. However, the diversity of the Cristamonadea remains understudied and underrepresented in the literature, comparatively few species have been formally described, and fewer still are associated with molecular sequence data^14–16^. As a result, the phylogeny of cristamonads is only poorly known, is dominated by environmental sequences from unidentified termite symbionts, and the underlying mechanisms and patterns underpinning their complicated morphological transitions remains unclear.

To further explore the diversity and phylogenetic positions of underrepresented parabasalian lineages, here we describe seven new species of cristamonad parabasalians found in the hindguts of lower termites, including two new species of the genus *Devescovina*^17^ from Ecuador and Peru, and three new species of the genus *Macrotrichomonas*^18^ from Peru. Additionally, we describe two species assigned to a new genus, *Runanympha* n. gen., also from Peru, and provide a molecular phylogeny of the class Cristamonadea based on their data from small subunit (SSU) rRNA sequences.

## Results and discussion

### Host collection and identification

*Calcaritermes temnocephalus*^19^ was collected near the Campoverde District of Peru and its morphological identification was confirmed by DNA barcoding (accession MF062149^20^). *Rugitermes laticollis*^21^, *Comatermes perfectus*^22^, and *Calcaritermes rioensis*^23^ were collected in Quito Ecuador, Tingo María, Peru, and the Huánuco Department of Peru, respectively, and were morphologically identified to the species level, and by DNA barcoding (accessions MF062147^20^, MT975287, and MT975289, respectively). However, there are no available comparison sequences for any of these species and so DNA barcoding could only be consistent with these morphological identifications (where sequences from other members of the genus were available). DNA barcoding was also used to rule out that these sequences were of any other barcoded species, as they did not share species-level similarity to any known sequence. The *Rugitermes laticollis* barcode was most similar to “*Rugitermes* sp. A TB-2014”, with 91.15% similarity (collection location: Petit Saut, French Guiana, accession number: KP026284^24^). *Comatermes perfectus* was most closely related to “*Kalotermes* sp. MM2013” with 87.47% similarity (collection location: Israel, accession: KC914322). *Calcaritermes rioensis* was most closely related to “*Calcaritermes nearcticus*” with 88.22% similarity (collection location: Florida, USA, accession: KJ438364^25^).

Lastly, an unidentified termite determined as representing a new species of *Rugitermes* was collected near Huánuco, Peru. Field identification and DNA barcoding both confirmed it to be a member of *Rugitermes*, but its morphology was not consistent with any known species. The most similar sequence to the barcode (accession MT975288) is “*Rugitermes* sp. A TB-2014” with a similarity of 89.81% (accession: KP026284^24^). Its formal description is in progress.

### Morphology of new Cristamonadea species

**Figure 1:**
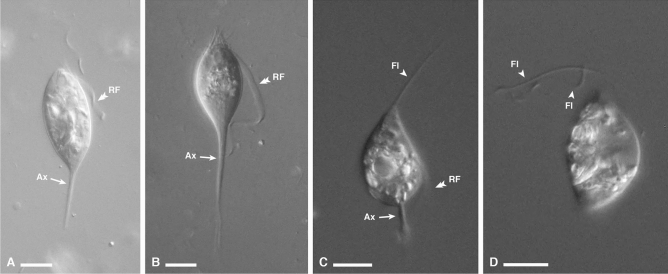
Differential interference contrast light micrographs of *Devescovina* specimens. (**A**,**B**) Images of *Devescovina sapara* from *Rugitermes laticollis* showing the overall body shape, with the recurrent flagellum and posteriorly protruding axostyle. (**C**,**D**) Images of *Devescovina aymara* from *Rugitermes* sp. showing the overall body shape, flagella, recurrent flagellum, and axostyle. Bars represent 10 μm. *RF* recurrent flagellum, *Ax* axostyle, *Fl* flagella.

**Figure 2:**
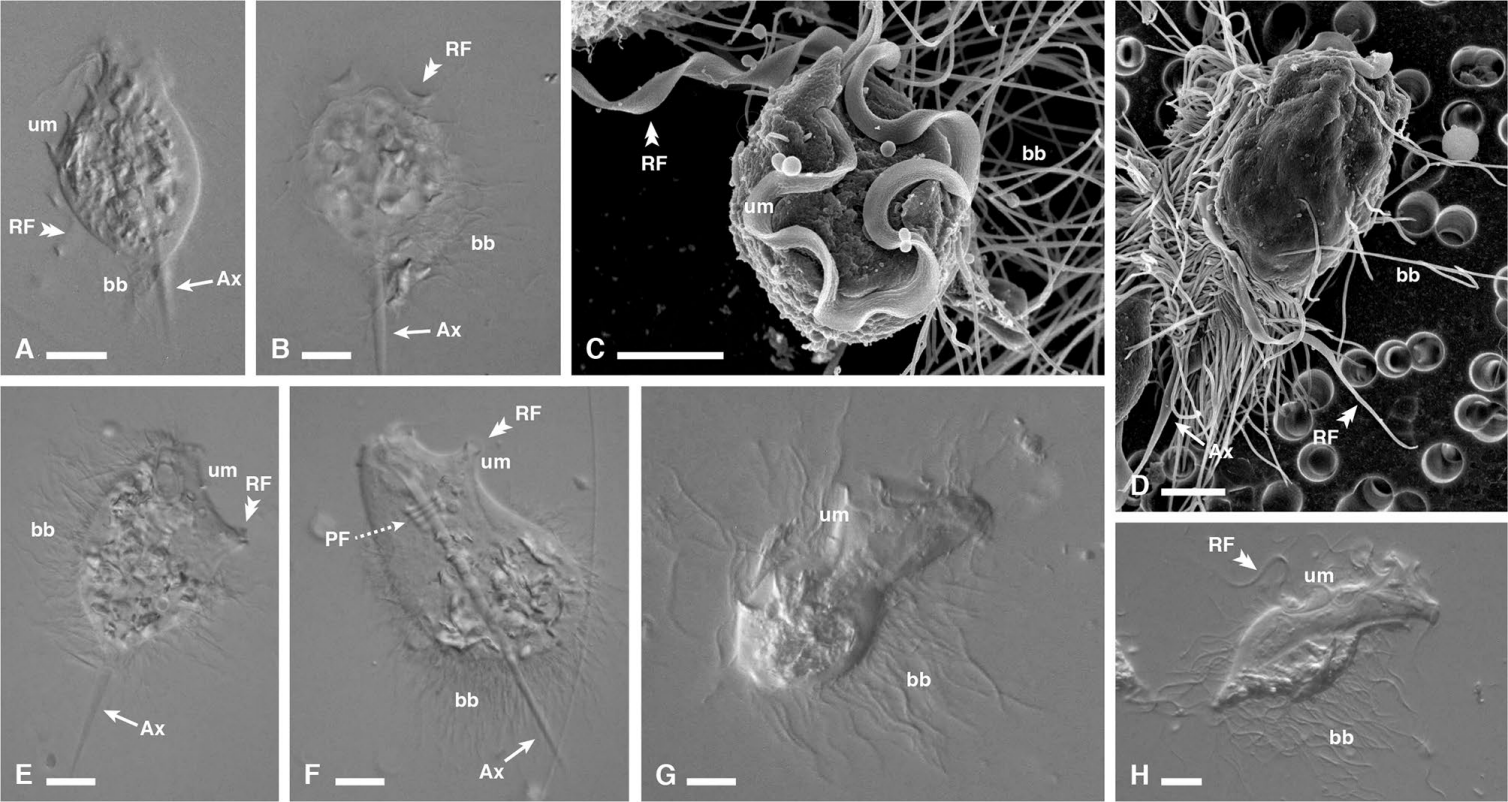
Light and electron micrographs of new *Macrotrichomonas* specimens. (**A**,**B**) Differential interference contrast (DIC) and (**C**,**D**) SEM images of *Macrotrichomonas ashaninka* from *Comatermes perfectus* showing the overall body shape, with the recurrent flagellum forming an undulating membrane where it adheres to the cell. Also visible are the axostyle and tufts of bacteria adhered to the cell surface. (**E**,**F**) DIC images of *Macrotrichomonas secoya* from *Calcaritermes temnocephalus* showing the overall body shape, with the recurrent flagellum forming an undulating membrane where it adheres to the cell. Also visible are bacterial tufts attached to the cell surface, and parabasal fibers coiled around the axostyle. (**G**,**H**) DIC images of *Macrotrichomonas yanesha* from *Calcaritermes rioensis* showing the overall body shape, with the recurrent flagellum forming an undulating membrane where it adheres to the cell. Also visible are tufts of long bacteria on the cell surface. *RF *recurrent flagellum, *um *undulating membrane, *Ax* axostyle, *bb* bacteria, *PF* parabasal fibers.

**Figure 3:**
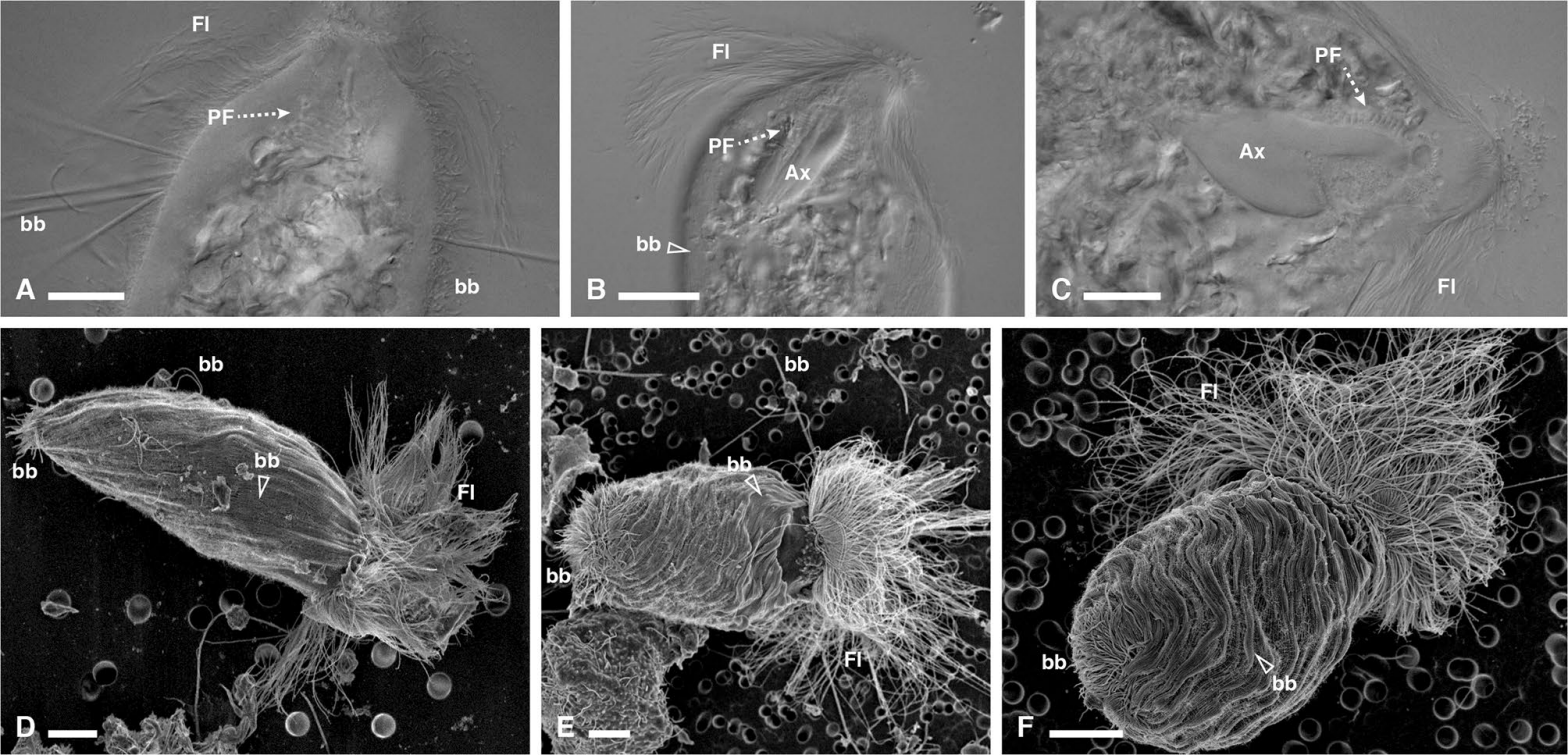
Light and electron micrographs of *R. illapa* specimens*.* (**A**–**C**) DIC and (**D**–**F**) SEM images of *Runanympha illapa* specimens from *Comatermes perfectus* showing the overall body shape, with the flagella, surface bacteria, axostyle, and spiralized parabasal fibers. *Fl *flagella, *bb* bacteria, *Ax* axostyle, *PF* parabasal fibers.

**Figure 4:**
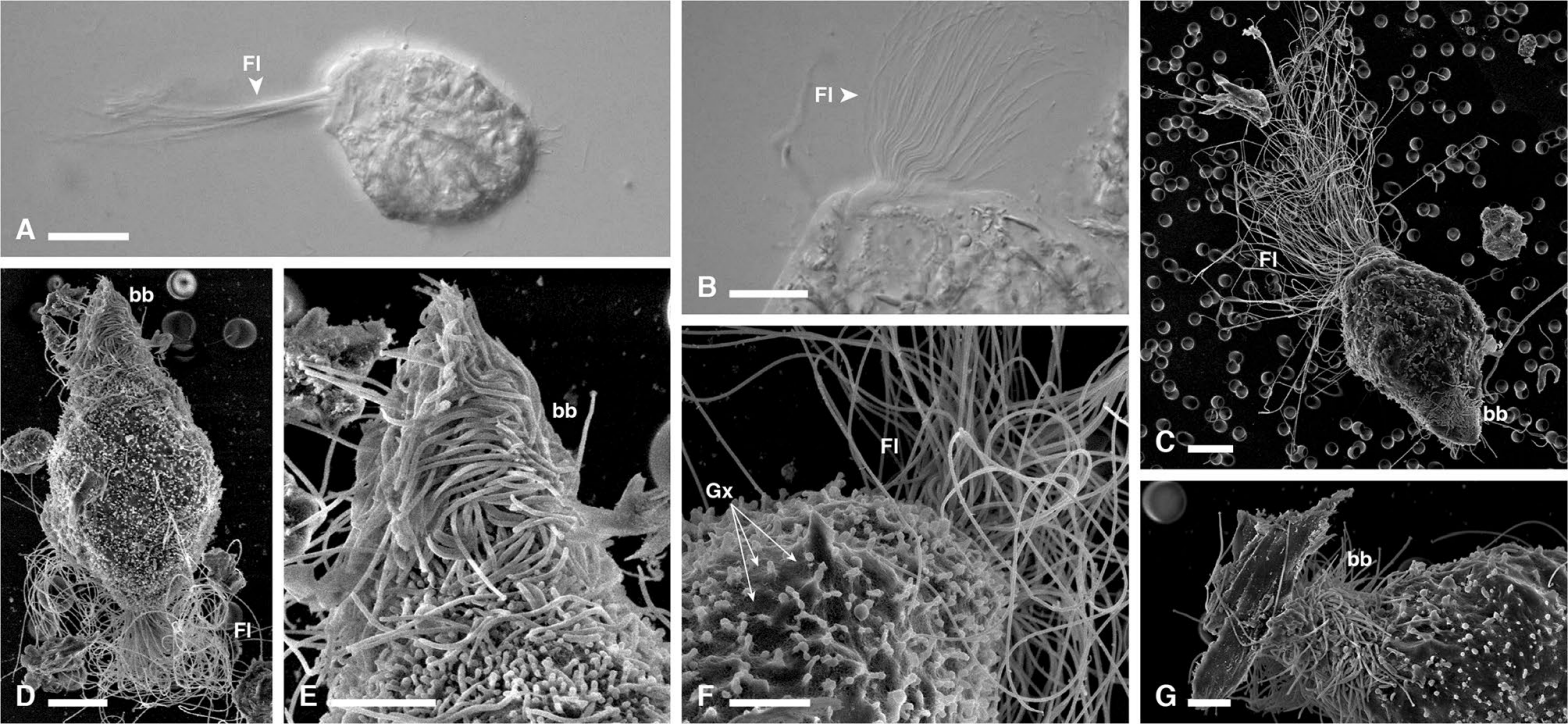
Light and electron micrographs of *R. pacha* specimens. (**A**,**B**) DIC and (**C**–**G**) SEM images of *Runanympha pacha* specimens from *Comatermes perfectus* showing the overall body shape, with flagella, tufts of surface bacteria on the posterior tip of the cell surface, axostyle, and glycocalyx. *Fl* flagella, *bb* bacteria, *Ax* axostyle, *Gx* glycocalyx.

In all five termite species, we observed and documented cells matching the description of known genera in the class Cristamonadea (Figs. 1, 2, 3, 4). All seven symbionts described below had cell bodies that were filled with wood particles, as well as prominent parabasal fibers visibly coiled around a thick axostyle—a feature distinctive to Cristamonadea. Also observed in the seven symbionts was a single large nucleus nested within the pelta—an extension of the axostyle. Specific details are recorded below.

*Devescovina sapara* n. sp. was observed in *Rugitermes laticollis* (Fig. 1A,B, and Supplementary Video), with an ovoid body shape and a long axostyle (almost as long as or longer than the cell body itself), which extended to the posterior end of the cell body, giving it an overall tapered appearance. The cell possessed a conspicuous long, thickened, and ribbon-like recurrent flagellum that was not adhered to the body. These are all features characteristic of the genus but not collectively found in other cristamonad genera (e.g., in various other genera the recurrent flagellum is just slightly thicker than normal, cord-like, shorter than the cell body, or forming an undulating membrane). Free anterior flagella were also observed: they had typical flagellar size and appearance, and were not counted but appeared consistent with the expected number for this genus of three. Mean cell size was as follows: length 31 μm (21–44 μm, n = 23), width 14 μm (11–18 μm). *Devescovina aymara* n. sp. from *Rugitermes* sp. (Fig. 1C,D, and Supplementary Video) was characterized by an obpyriform-shaped body (a pointed anterior end and a more spherical middle region) with the axostyle extending out at the posterior end of the cell. These flagellates also shared the other noted features typical of the genus such as the long, thick, free, ribbon-like recurrent flagellum and a few free anterior flagella. Mean cell size was: length 24 μm (18–28 μm, n = 6), width 14 μm (12–16 μm). Both of these *Devescovina* species were morphologically distinguished from the genus *Metadevescovina* (which has been historically difficult to differentiate) by their elongated cell shapes as opposed to the stout bodies of *Metadevescovina*.

*Macrotrichomonas ashaninka* n. sp. from *Comatermes perfectus* (Fig. 2A,D, and Supplementary Video) had an ovoid-to-pyriform-shaped body with a slightly tapered anterior end and a distinctive undulating membrane that spanned the entire length of the cell body, and sometimes extended slightly beyond its attachment to the body, where it appeared as a thickened and band-like flagellum. This morphological characteristic distinguishes *Macrotrichomonas* from most other cristamonad genera. Furthermore, these flagellates were distinguished from the genus *Gigantomonas,* which share many of the same morphological characteristics, due to the presence of a posteriorly tapering axostyle with a pointed end and with a posterior projection. Additionally, parabasal fibers are spiraled around the axostyle, which is not a feature of the genus *Gigantomonas*. Tufts of surface bacteria extending perpendicularly from the cell body were also visible. Mean cell size was: length 39 μm (34–43 μm, n = 6), width 26 μm (19–29 μm). *Macrotrichomonas secoya* n. sp. from *Calcaritermes temnocephalus* (Fig. 2E,F, and Supplementary Video) possessed an ovoid-shaped body with a posteriorly protruding axostyle, and an undulating membrane which spanned approximately half of the length of the cell body. Its cell was covered by large, elongated surface bacteria oriented perpendicularly to the cell body. Mean cell size was: length 54 μm (46–67 μm, n = 18), width 40 μm (32–54 μm). *Macrotrichomonas yanesha* n. sp. from *Calcaritermes rioensis* (Fig. 2G,H, and Supplementary Video) was generally oblong in shape and was also characterized by a thick undulating membrane, which spanned approximately half of the length of the cell body and posteriorly projecting axostyle. Large tufts of long, perpendicularly-oriented surface bacteria (about as long as the width of the cell body) were present as well. Mean cell size was: length 59 μm (53–62 μm, n = 5), width 27 μm (24–32 μm). *Macrotrichomonas* symbionts can not presently be distinguished morphologically from the genus *Macrotrichomonoides*^26^, and so this genus will be discussed below.

In addition to these flagellates with clear morphological similarities to well-described genera, we also observed large and complex “hypermastigote” flagellates that did not obviously fit the description of any known genera, and are here described as a new genus, *Runanympha*. Overall, these flagellates shared similarities in gross body plan with joeniids, a group of parabasalians that was historically recognized as a lineage of hypermastigotes, but has since proven to be taxonomically complex, and likely polyphyletic. Indeed, the relationships between major subgroups of the cristamonads are generally so poorly known that the most recent classification^1^ has simply lumped the entire order into a single family, Joeniidae. But molecular data from described species of joeniids are very rare, and what data do exist fail to support the monophyly of different genera. These various “joeniids” are characteristically large cells with a single nucleus and axostyle, and hundreds to thousands of flagella organized on large distinctively shaped plates. We observed two flagellates of very different appearance from one another sharing these overall features in *Comatermes perfectus*. The parabasal fibers of both *Runanympha* flagellates formed unbranched coils around the axostyle, which is also typical of *Placojoenia* and *Projoenia,* but neither of which shares distinguishing features with both *Runanympha* types. *Runanympha illapa* n. sp. (Fig. 3, and Supplementary Video) had a large, oblong body with a prominent tuft of motile flagella emerging from a curved plate at the anterior end of the cell. The flagellar tuft encompassed the entire apex of the cell unlike the small u-shaped tuft of flagella found in *Placojoenia*. The cell exterior was coated with a layer of short, perpendicularly-oriented bacteria, long perpendicularly-oriented bacteria that were sparser, as well as long bacteria situated in parallel to the cell axis creating striations across the cell surface. Mean cell size was: length 123 μm (99–150 μm, n = 9), width 62 μm (38–91 μm). *Runanympha pacha* n. sp. was also observed in the same host, (Fig. 4, and Supplementary Video) and although it also possessed an oblong-shaped body, it was markedly smaller (about half the size), and the tuft of motile flagella emerging from a plate were packed more closely together in a bundle at the anterior tip of the cell similar to the genus *Placojoenia*. However, *Runanympha pacha* lacked the numerous cytoplasmic plates of *Placojoenia* that are normally visible in the ectoplasm. The cell exterior was characterized by long bacteria situated in parallel to the cell surface and a glycocalyx. Mean cell size was: length 65 μm (46–96 μm, n = 28), width 52 μm (30–94 μm).

### Molecular phylogeny

To determine the relationships between these new species and other cristamonads, we sequenced the small subunit rRNA gene from single isolated cells and small pools of isolated cells. Sequences from cells of any given species shared, as expected, high mean pairwise identities (96.8–99.2%). All mean pairwise identity values are presented in Supplementary Table S1. A representative sequence was selected for each taxon (the complete sequence closest to the consensus) and used for further phylogenetic analyses. New sequences were submitted to GenBank under the accessions MT975290–MT975296.

**Figure 5:**
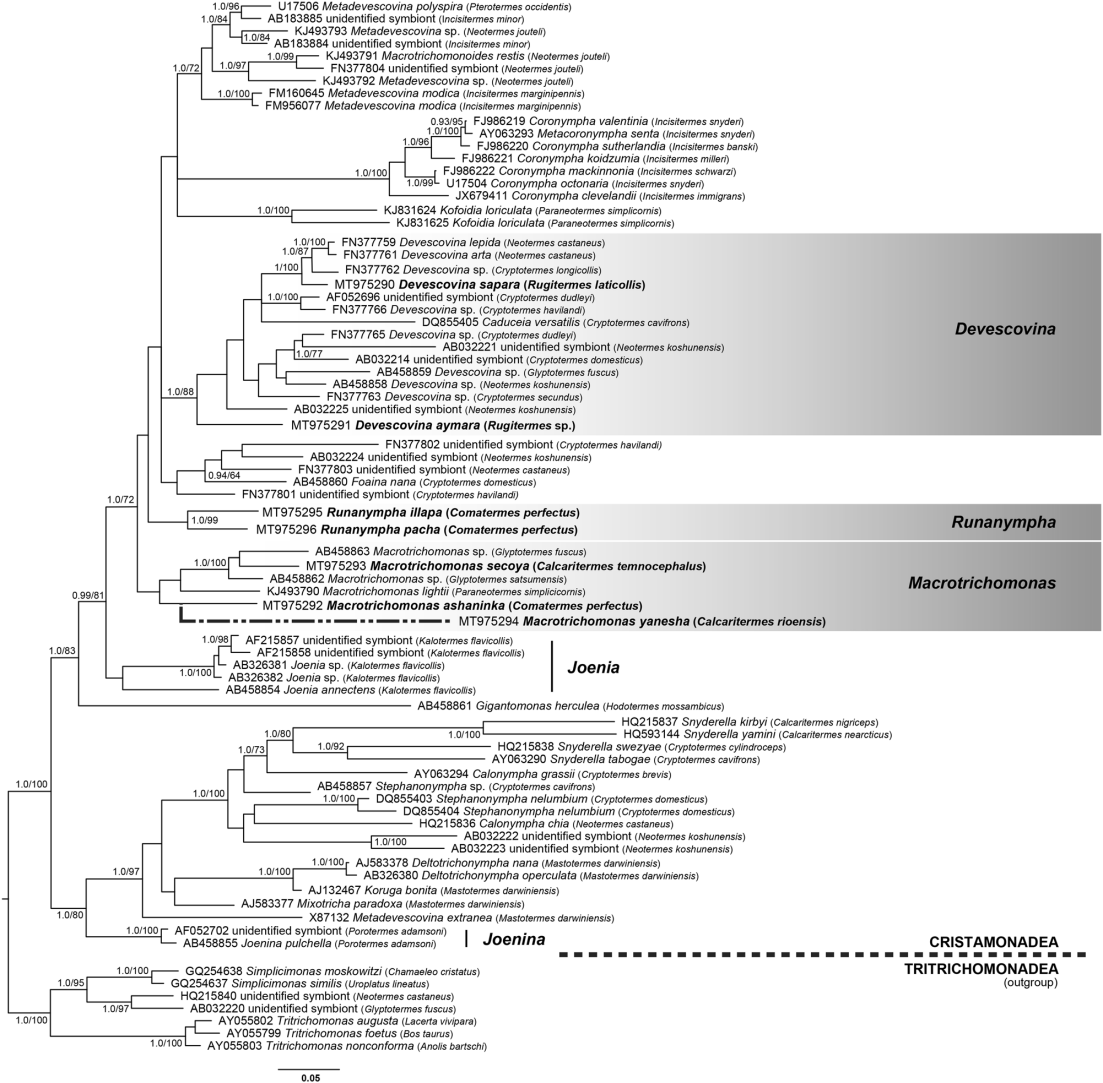
Bayesian phylogeny of SSU rRNA sequences of the class Cristamonadea (1528 sites). The tree is rooted with sequences from the closest known relatives to the Cristamonadea, members of the class Tritrichomonadea. Sequences obtained in this study are in bold type. Taxon names include GenBank accession numbers and the name of the termite host. Numerical values at nodes indicate Bayesian posterior probability (where greater than 0.90) and % ML bootstrap support (out of 1000 replicates, where in agreement with the Bayesian topology and greater than 60%). The non-uniform dashed line represents the manual addition of *M. yanesha* to the tree after analyses. Sequences assigned to or likely belonging to the joeniid genera *Joenia* and *Joenina* are denoted by a thick vertical bar. Tree figures were generated using the FigTree software v1.4.4 (http://tree.bio.ed.ac.uk/software/figtree/) and edited in Adobe Illustrator (https://adobe.com/products/illustrator).

The SSU rRNA gene sequence from *Macrotrichomonas yanesha* from *Calcaritermes rioensis* was extremely divergent: the branch separating it from other *Macrotrichomonas* sequences was 3.5 times longer than for any other *Macrotrichomonas* sequence. Although it branches where it is expected to based on morphology (with other members of the genus), inclusion of this sequence had a substantial impact on support values throughout the tree in both Bayesian and maximum likelihood (ML) analyses (Supplementary Fig. S1). Therefore, analyses were carried out both including and excluding this sequence (Fig. 5, and Supplementary Fig. S1). When excluded, support values generally increased but the overall topology changed very little (4 nodes in the ML phylogeny, and 1 node in the Bayesian phylogeny). Although the phylogenetic placement of this sequence is therefore uncertain, the cell possesses all the morphological characteristics expected of the genus *Macrotrichomonas*, and its phylogenetic position with other members of that genus suggests this is simply a species with a divergent rRNA gene and we therefore have no basis to question its assignment to the genus *Macrotrichomonas*.

In all analyses, *Macrotrichomonas secoya* branches with other members of that genus, confirming their identification and supporting their designation as distinct species. Similarly, *Macrotrichomonas ashaninka* also branches with the other members of the genus, although in this case lacking statistical support. (Fig. 5). However, as the cell possesses the morphological characteristics expected of the genus *Macrotrichomonas,* there is currently no evidence to question its assignment to the genus.

Both new *Devescovina* species also branch with the clade that also includes all other currently identified members of the genus; several sequences identified as “*Devescovina* sp.”, as well as the genus *Caduceia*^11,27^ and sequences of “unidentified symbionts”^28,29^. However, whereas *Devescovina sapara* from *Rugitermes laticollis* branches within a clade of other described *Devescovina* species, *Devescovina aymara* from *Rugitermes* sp. is notably deep-branching, falling as the sister to the entire clade in our phylogeny, although this position lacks statistical support.

The two *Runanympha* symbionts from *Comatermes perfectus* branched together with strong support, but their rRNA gene sequences did not share a high degree of identity, consistent with their very distinct morphology that they represent distinct species. Although these are both morphologically “joeniid-like”, they do not branch with any currently sampled “joeniid”, which includes the genera *Joenia*, *Joenina*, and *Joenoides* (the latter of which is also highly divergent and trees are shown both with and without this sequence: Fig. 5, and Supplementary Fig. S2). Morphologically they have similarities to the genus *Joenia,* however one of the few “joeniid” sequences that has been characterized is annotated as being from the type species from the type host of the genus *Joenia*: *Joenia annectens* from *Kalotermes flavicollis*^30,31^. The *J. annectens* SSU gene sequence falls within a clade of other *K. flavicollis* symbionts inferred to also be joeniids, which is not closely related to the *Runanympha* clade (Fig. 5). Molecular data are unavailable from several other “joeniid” genera, including *Placojoenia* and *Projoenia.*

### Taxonomic considerations

In early taxonomic studies of termite symbionts based on light microscopy, there was a tendency by some authors to identify the same symbiont in multiple termite hosts, while others tended to assume different hosts had different symbionts^1,2,6,11,30,32^. Distinguishing symbionts at the species level based only on morphology in light microscopy is obviously problematic, so more recent work has been based largely on molecular data. Here symbionts are generally concluded to be highly host-specific, and switching between different termite hosts is very rare, as is having the same symbiont in two different hosts^1,4,5,25^. This conclusion is often used to explain high-level patterns. For example, termite hosts within the Kalotermitidae family have been shown to harbour a large diversity of cristamonads but few trichonymphids, whereas the presence of cristamonads in other termite families is relatively uncommon^25^. But the pattern of host-symbiont specificity has only been tested in a few cases using hosts that are extremely closely-related (e.g., several members of the same genus). Here, the conclusions based on molecular data have been less consistent. For example, one study based on four species of *Isotermes* concluded the symbionts maintain a degree of sequence divergence similar to that of their hosts^33^, overall supporting the conclusion that symbionts are host-specific. However, a more recent study of three species of *Zootermopsis* that are extremely close relatives (i.e. the animals are difficult to differentiate based on morphology) that also included several populations and data from all species of symbionts revealed a more complex pattern. Here, host-speciation did correlate with speciation for some symbionts, but other symbionts were not found in all hosts, and others lacked the degree of sequence variation of their hosts, suggesting incomplete co-cladogenesis^34^. This does not suggest that symbionts move between hosts at high frequencies as a rule, but does open the possibility that either the rates of change in the gene examined are uneven or that symbionts are exchanged between closely-related hosts in overlapping geographic locations^34^. Taken together these studies are all consistent with the conclusion that symbionts in different hosts are most likely to represent different species as well, with a note of caution when dealing with hosts that are especially closely related.

Consequently, the two new *Devescovina* symbionts described here are inferred to represent new species of *Devescovina* because no *Devescovina* species has been formally described from either host. In the case of *Rugitermes laticollis* only one symbiont has been formally described (belonging to the genus *Trichonympha*^35^), and the other host appears itself to be a new species^6,20^. At the molecular level, neither symbiont is closely related to any existing species with molecular data. Formally, the position of *Caduceia* would make the genus *Devescovina* paraphyletic if one includes the new *R. laticollis* symbiont in the genus. However, without more information from the many “*Devescovina* sp.” sequences in the tree, a better resolved tree based on more genes, and a reexamination of *Caduceia* (a genus also known as “rubberneckia” with a confused taxonomic history^36,37^), we conclude the most conservative action is to not create a new genus for a species without significant variation from typical *Devescovina* morphology in what appears to be an otherwise monophyletic molecular clade.

Similarly, the three described species of *Macrotrichomonas* are all inferred to be new species because no such symbiont has been described in any of the host species (for *Comatermes perfectus* and *Calcaritermes rioensis* no flagellates have been formally described, and from *Calcaritermes temnocephalus* only *Trichonympha* has been reported^20^), and none is found to be identical (or close) to any known species with molecular data.

Lastly, the remaining two specimens from *Comatermes perfectus* characterized here are assigned to new species in a new genus. To date, no symbionts have been reported from any member of the host genus *Comatermes*, and the SSU rRNA genes from both are phylogenetically distinct. In addition, although these symbionts have at least superficial morphological similarities to the genus *Joenia*, the phylogeny shows they do not branch with the type species for that genus, *Joenia annectens* from the type host *Kalotermes flavicollis*^30^*.* Based on this, and on the known polyphyly of “joeniids” as well as the lack of any clear evidence that they belong to any described genus, we conclude these symbionts represent not only new species but also a new genus. We do however acknowledge that the molecular data from joeniids are sparse, and that future sampling may reveal a relationship to one of the few described genera for which no molecular data has been sampled, at which time transferring these species to that genus will have to be reevaluated.

### Taxonomic summary

#### Assignment

Eukaryota; Parabasalia (Honigberg, 1973); Cristamonadea (Brugerolle & Patterson, 2001); Cristamonadida (Brugerolle & Patterson, 2001); Joeniidae (Janicki, 1915); *Devescovina* (Foà, 1905).

### *Devescovina sapara* n. sp. Singh and Keeling

urn:lsid:zoobank.org:act:BFBBB33A-22DA-4B7C-B990-602CAD5358C6

**Type host:** *Rugitermes laticollis* (Isoptera, Kalotermitidae: barcode MF062147^20^).

**Type locality:** Parque La Carolina, Quito, Ecuador (0.18845 S 78.48595 W).

**Host collection:** University of Florida termite collection, accession number EC1465. Collectors: Mullins, Scheffrahn and Křeček^38^.

**Description:** Parabasalian flagellate with morphological characteristics of the genus *Devescovina*. Mean cell size is: length 31 μm (21–44 μm, n = 23), and width 14 μm (11–18 μm). Ovoid cell body shape. Long axostyle protruding outwardly from the posterior end of the cell body. Long, voluminous, ribbon-like recurrent flagellum. Found in the hindgut of *Rugitermes laticollis*. Distinct SSU rRNA gene sequence.

**Holotype:** Specimen in Fig. 1B of the present publication.

**Gene sequence:** SSU rRNA gene GenBank accession number MT975290.

**Etymology:** This species is named for the Sápara people of South America.

#### Assignment

Eukaryota; Parabasalia (Honigberg, 1973); Cristamonadea (Brugerolle & Patterson, 2001); Cristamonadida (Brugerolle & Patterson, 2001); Joeniidae (Janicki, 1915); *Devescovina* (Foà, 1905).

### *Devescovina aymara* n. sp. Singh and Keeling

urn:lsid:zoobank.org:act:BF39676F-B235-4687-AF2A-4FF819BD0352

**Type host:** *Rugitermes* sp. (Isoptera, Kalotermitidae: barcode MT975288).

**Type locality:** Huánuco, Peru (9.87712 S 76.16408 W).

**Host collection:** University of Florida termite collection, accession number PU990. Collectors: Carrijo, Constantino, Chase, Křeček, Kuswanto, Mangold, Mullins, Nishimura, and Scheffrahn.

**Description:** Parabasalian flagellate with morphological characteristics of the genus *Devescovina*. Mean cell size is: length 24 μm (18–28 μm, n = 6), and width 14 μm (12–16 μm). Obpyriform-shaped with a protruding axostyle. Thick recurrent flagellum. Packed with wood particles. Found in the hindgut of *Rugitermes* sp. Distinct SSU rRNA gene sequence.

**Holotype:** Specimen in Fig. 1C of the present publication.

**Gene sequence:** SSU rRNA gene GenBank accession number MT975291.

**Etymology:** This species is named for the Aymara people of South America.

#### Assignment

Eukaryota; Parabasalia (Honigberg, 1973); Cristamonadea (Brugerolle & Patterson, 2001); Cristamonadida (Brugerolle & Patterson, 2001); Joeniidae (Janicki, 1915); *Macrotrichomonas* (Grassi, 1917).

### *Macrotrichomonas ashaninka* n. sp. Singh and Keeling

urn:lsid:zoobank.org:act:2795074E-17A0-4766-A338-79B260F4B2CC

**Type host:** *Comatermes perfectus* (Isoptera, Kalotermitidae: barcode MT975287).

**Type locality:** Tingo María, Peru (9.14974 S 75.99233 W).

**Host collection:** University of Florida termite collection, accession number PU944. Collectors: Carrijo, Constantino, Chase, Křeček, Kuswanto, Mangold, Mullins, Nishimura, and Scheffrahn.

**Description:** Parabasalian flagellate with morphological characteristics of the genus *Macrotrichomonas*. Mean cell size is: length 39 μm (34–43 μm, n = 6), and width 26 μm (18–29 μm). Pyriform cell body with a tapered anterior end. Ribbon-like recurrent flagellum adhered to an undulating membrane along the entire length of the cell body. Tufts of bacteria on the cell exterior extending perpendicularly from the cell body. Found in the hindgut of *Comatermes perfectus*. Distinct SSU rRNA gene sequence.

**Holotype:** Specimen in Fig. 2A of the present publication.

**Gene sequence:** SSU rRNA gene GenBank accession number MT975292.

**Etymology:** This species is named for the Asháninka people of South America.

#### Assignment

Eukaryota; Parabasalia (Honigberg, 1973); Cristamonadea (Brugerolle & Patterson, 2001); Cristamonadida (Brugerolle & Patterson, 2001); Joeniidae (Janicki, 1915); *Macrotrichomonas* (Grassi, 1917).

### *Macrotrichomonas secoya* n. sp. Singh and Keeling

urn:lsid:zoobank.org:act:4C7EFB5C-B055-4479-A51A-661128006120

**Type host:** *Calcaritermes temnocephalus* (Isoptera, Kalotermitidae: barcode MF062149^20^).

**Type locality:** Ucayali Department, Peru (8.60854 S 74.93628 W).

**Host collection:** University of Florida termite collection, accession number PU512. Collectors: Carrijo, Constantino, Chase, Křeček, Kuswanto, Mangold, Mullins, Nishimura, and Scheffrahn.

**Description:** Parabasalian flagellate with morphological characteristics of the genus *Macrotrichomonas*. Mean cell size is: length 54 μm (46–67 μm, n = 18), and width 40 μm (32–54 μm). Ovoid cell body. Recurrent flagellum adhered to an undulating membrane that spans half the length of the cell body. Axostyle protruding out far from posterior end of the cell body. Large, elongated bacteria adhered perpendicularly to the cell exterior along the entire length of the cell body. Filled with wood either entirely or saturated in the posterior region only. Found in the hindgut of *Calcaritermes temnocephalus*. Distinct SSU rRNA gene sequence.

**Holotype:** Specimen in Fig. 2F of the present publication.

**Gene sequence:** SSU rRNA gene GenBank accession number MT975293.

**Etymology:** This species is named for the Secoya people of South America.

#### Assignment

Eukaryota; Parabasalia (Honigberg, 1973); Cristamonadea (Brugerolle & Patterson, 2001); Cristamonadida (Brugerolle & Patterson, 2001); Joeniidae (Janicki, 1915); *Macrotrichomonas* (Grassi, 1917).

### *Macrotrichomonas yanesha* n. sp. Singh and Keeling

urn:lsid:zoobank.org:act:3D73E79E-4F3A-4F7C-B079-C7805F934807

**Type host:** *Calcaritermes rioensis* (Isoptera, Kalotermitidae: barcode MT975289).

**Type locality:** Huánuco Department, Peru (9.70524 S 75.0169 W).

**Host collection:** University of Florida termite collection, accession number PU245. Collectors: Carrijo, Constantino, Chase, Křeček, Kuswanto, Mangold, Mullins, Nishimura, and Scheffrahn.

**Description:** Parabasalian flagellate with morphological characteristics of the genus *Macrotrichomonas*. Mean cell size is: length 59 μm (52–62 μm, n = 5), and width 27 μm (24–32 μm). Oblong cell body shape. Recurrent flagellum attached to an undulating membrane that spans half the length of the cell body. Many tufts of long bacteria adhered to the cell exterior and oriented perpendicular to the body. Wood particles found either throughout the cell body or in the posterior region only. Found in the hindgut of *Calcaritermes rioensis*. Distinct SSU rRNA gene sequence.

**Holotype:** Specimen in Fig. 2H of the present publication.

**Gene sequence:** SSU rRNA gene GenBank accession number MT975294.

**Etymology:** This species is named for the Yanesha’ (or Amuesha) people of South America.

#### Assignment

Eukaryota; Parabasalia (Honigberg, 1973); Cristamonadea (Brugerolle & Patterson, 2001); Cristamonadida (Brugerolle & Patterson, 2001); Joeniidae (Janicki, 1915).

### *Runanympha* n. g. Singh and Keeling

urn:lsid:zoobank.org:act:1D4C7F4B-4738-415D-BCDA-AC9FA195CF4D

**Type species:** *Runanympha illapa* (Fig. 3B).

**Type host:** *Comatermes perfectus* (Isoptera, Kalotermitidae: barcode MT975287).

**Description:** Large parabasalian mononucleate flagellate with robust parabasal fibers coiled around the trunk of a single axostyle. Tuft of flagella emerge from an anterior plate of basal bodies. Filled with wood particles.

**Etymology:** The genus is named for the Quechua people, indigenous peoples of South America (originating in Peru—where these species were found) that speak the Quechua language. The Quechua word for a Quechua speaker is “runa”. Pronounced “roo-nah-nim-fah”.

#### Assignment

Eukaryota; Parabasalia (Honigberg, 1973); Cristamonadea (Brugerolle & Patterson, 2001); Cristamonadida (Brugerolle & Patterson, 2001); Joeniidae (Janicki, 1915); *Runanympha* (Singh & Keeling).

### *Runanympha illapa* n. sp. Singh and Keeling

urn:lsid:zoobank.org:act:CC985A2C-10D7-4CBB-B2F9-714E364FBEEC

**Type host:** *Comatermes perfectus* (Isoptera, Kalotermitidae: barcode MT975287).

**Type locality:** Tingo María, Peru (9.14974 S 75.99233 W).

**Host collection:** University of Florida termite collection, accession number PU944. Collectors: Carrijo, Constantino, Chase, Křeček, Kuswanto, Mangold, Mullins, Nishimura, and Scheffrahn.

**Description:** Parabasalian flagellate with morphological characteristics of the genus *Runanympha*. Mean cell size is: length 123 μm (99–150 μm, n = 9), and width 62 μm (38–91 μm). Large, oblong body with a big tuft of motile flagella spread out along the anterior end of the cell. Wood particles either filling up the entire cell or localized to the centre. Short bacteria protruding perpendicularly along the length of the cell. Longer bacteria situated parallel to the cell surface creating striation patterns across the cell body. Found in the hindgut of *Comatermes perfectus*. Distinct SSU rRNA gene sequence.

**Holotype:** Specimen in Fig. 3B of the present publication.

**Gene sequence:** SSU rRNA gene GenBank accession number MT975295.

**Etymology:** This species is named for Illapa, the ancient Incan divinity of thunder, lightning, and rain.

#### Assignment

Eukaryota; Parabasalia (Honigberg, 1973); Cristamonadea (Brugerolle & Patterson, 2001); Cristamonadida (Brugerolle & Patterson, 2001); Joeniidae (Janicki, 1915); *Runanympha* (Singh & Keeling).

### *Runanympha pacha* n. sp. Singh and Keeling

urn:lsid:zoobank.org:act:EDC9240B-07A4-4A27-8064-6CE29208A49A

**Type host:** *Comatermes perfectus* (Isoptera, Kalotermitidae: barcode MT975287).

**Type locality:** Tingo María, Peru (9.14974 S 75.99233 W).

**Host collection:** University of Florida termite collection, accession number PU944. Collectors: Carrijo, Constantino, Chase, Křeček, Kuswanto, Mangold, Mullins, Nishimura, and Scheffrahn.

**Description:** Parabasalian flagellate with morphological characteristics of the genus *Runanympha*. Mean cell size is: length 65 μm (46–96 μm, n = 28), and width 52 μm (30–94 μm). Oblong cell body shape. A tuft of motile flagella packed closely together in a bundle at the anterior tip. Wood particles either filling up the entire cell or localized to the centre. Long bacteria situated parallel to the cell surface creating striation patterns across the cell body. Glycocalyx surrounding the cell membrane. Found in the hindgut of *Comatermes perfectus*. Distinct SSU rRNA gene sequence.

**Holotype:** Specimen in Fig. 4C of the present publication.

**Gene sequence:** SSU rRNA gene GenBank accession number MT975296.

**Etymology:** This species is named for Pachamama, the ancient Incan divinity of the earth.

## Methods

### Host termite collection and barcoding

*Comatermes perfectus* was collected 15 km north of Tingo María, Peru on May 31, 2014. *Calcaritermes temnocephalus* and *Calcaritermes rioensis* were collected 20 km SW of the Ucayali Department of Peru and 16 km north of Ciudad Constitución on May 28, 2014 and May 27, 2014 respectively. *Rugitermes laticollis* was collected in Parque La Carolina, Quito, Ecuador on June 4, 2011 at 2822 m above sea level and another *Rugitermes* that could only be morphologically identified to the genus level was collected 10 km NE of Huánuco, Peru on June 1, 2014. All specimens have been deposited into the University of Florida termite collection under the accessions PU944, PU512, PU245, EC1465, and PU990, respectively. Termite hosts were identified by morphological criteria by light microscopy and then maintained in conical tubes in the lab at room temperature with wood from their habitats. Termite hosts were also identified through DNA barcoding using the mitochondrial large subunit (mtLSU) ribosomal RNA gene. DNA was extracted from termite bodies using the Masterpure Complete DNA and RNA Purification Kit (Epicentre) and the mtLSU rRNA gene was amplified with the primers LR-N-13398 5′-CGC CTG TTT ATC AAA AAC AT-3′^39^ and LR-J-13017 5′-TTA CGC TGT TAT CCC TAA-3′^40^. PCR conditions included a 3 min denaturation at 95 °C followed by 30 cycles of 95 °C for 30 s, 50 °C for 30 s, and 72 °C for 1 min, then an additional 7 min at 72 °C. PCR products were purified using the QIAquick PCR purification kit (Qiagen) and sequenced with a BigDye Terminator v. 3.1 kit (Applied Biosystems). All sequences were deposited in GenBank under the accessions MF062147^20^, MF062149^20^, MT975287, MT975288, and MT975289.

### Microscopy

Termites were dissected and hindgut contents were suspended in Trager’s medium U^41^. Symbionts were observed with an Axioplan 2 DIC microscope (Zeiss) and multiple cells of each symbiont species were filmed with a 3CCD HD video camera XL H1S (Canon). Cell sizes and cell shapes were estimated using multiple documented cells although in some cases the size range was considerable since many cells were observed to deviate from the norm such as in *Runanympha illapa* and *Runanympha pacha*. Single cell isolations for molecular characterization were performed using an Axiovert 200 inverted microscope (Zeiss).

For SEM images, termite hindguts were removed by forceps and suspended in Trager’s medium U^41^. Gut contents were transferred by a glass micropipette to 2% glutaraldehyde (in Trager’s medium U) on ice. A 3 μm polycarbonate membrane filter was placed within a Swinnex filter holder (Millipore Corp., Billerica, MA). Gut contents were filtered onto the membrane using a syringe with distilled water, and the holder was placed in a small beaker (4 cm diam. and 5-cm-tall) that was filled with distilled water. Ten drops of 1% OsO_4_ were added to the opening of the filter holder, and the samples were post fixed on ice for 30 min. The syringe was used to slowly run distilled water over all samples. A graded series of ethanol washes (30%, 50%, 75%, 85%, 95%, and 100%) were then used to dehydrate the fixed cells using the syringe system. Following dehydration, the polycarbonate membrane filters containing the gut contents were transferred from the Swinnex filter holders into an aluminum basket submerged in 100% ethanol in preparation for critical point drying with CO_2_. The dried polycarbonate membrane filters containing the gut contents were mounted on aluminum stubs, sputter coated with 5 nm gold and viewed/photographed under a Hitachi S4700 scanning electron microscope at 5 kV.

### Single cell isolation, sequencing

Single cells or pools of cells were isolated with a glass micropipette and rinsed three times for DNA extraction using the Masterpure Complete DNA and RNA Purification Kit (Epicentre). SSU (18S) rRNA genes were amplified from the purified DNA using the eukaryotic specific primers PFI 5′-TGC GCT ACC TGG TTG ATC CTG CC-3′^42^ and FAD4 5′-TGA TCC TTC TGC AGG TTC ACC TAC-3′^43,44^. PCR conditions included a 3-min denaturation at 95 °C followed by 30 cycles of 95 °C for 30 s, 55 °C for 30 s, and 72 °C for 1 min 30 s, then an additional 7 min at 72 °C. Products were purified, cloned into the pCR2.1 vector using the TOPO-TA cloning kit (Invitrogen), and sequenced on both strands with BigDye Terminator v3.1 (Applied Biosystems). Multiple clones were sequenced from each isolated single cell.

### Phylogenetic analysis

After sequencing, low quality sequences that were incomplete or contained many unresolved bases were discarded from the analyses. The following number of sequences from clones were kept for initial analyses: two clones from one cell of *Devescovina sapara*, two clones from one single cell and two clones from a pool of twenty cells of *Devescovina aymara*, eight clones from five single cells of *Macrotrichomonas ashaninka*, three clones from two single cells of *Macrotrichomonas secoya*, three clones from two single cells of *Macrotrichomonas yanesha*, three clones from two single cells of *Runanympha illapa*, and three clones from two single cells of *Runanympha pacha*. In all cases, the remaining sequences from a given species were 96.8–99.2% identical (mean pairwise identities). One representative sequence per taxon was used for phylogenetic analyses and submitted to GenBank under accessions MT975290–MT975296. However, due to the high divergence of the representative sequence for *Macrotrichomonas yanesha*, all analyses were redone without this sequence.

The representative sequences were aligned with 63 available sequences from the class Cristamonadea and 7 sequences from the closely related class Tritrichomonadea using MAFFT v 7.212 (setting: —linsi)^45^. Highly variable regions were removed using trimAl v 1.2 (setting: —gt 0.3)^46^ resulting in a final alignment of 76 taxa and 1528 positions (or 77 taxa and 1527 positions when the divergent *Macrotrichomonas* was included). Maximum likelihood analyses were performed with IQ-TREE multicore v 1.5.5^47^ using the model GTR + R5 as recommended by the BIC criterion through modelfinder (setting: —m MFP)^48^ and ML support was assessed from 1000 standard non-parametric bootstrap replicates. Bayesian analyses were performed with MrBayes v 3.2.2^49^. Using a GTR + Γ model, four chains were sampled every 100 generations from three runs for 1,000,000 generations and diagnostics were run every 5000 generations. 25% of the trees were discarded as burnin and after 1,000,000 generations, the average standard deviation of the split frequencies from the three runs was 0.008128.

## Supplementary Information

Supplementary Information.

Supplementary Video 1.
