# Supplementary material for: Characterization of new cristamonad species from kalotermitid termites including a novel genus, Runanympha
Source: Sci Rep. 2021 Mar 31;11:7270. doi: 10.1038/s41598-021-86645-w (PMC8012604; doi:10.1038/s41598-021-86645-w)
Supplement: Supplementary file 1 — Supplementary Information. [file 41598_2021_86645_MOESM1_ESM.pdf]

Supplementary Material

Characterization of new cristamonad species from kalotermitid termites including a novel genus, *Runanympha*.

Racquel A. Singh[1], Vittorio Boscaro[1], Erick R. James[1], Anna Karnkowska[1], Martin Kolisko[1], Gregory S. Gavelis[1], Noriko Okamoto[1], Javier del Campo[1], Rebecca Fiorito[1], Elisabeth Hehenberger[1], Nicholas A. T. Irwin[1], Varsha Mathur[1], Rudolf H. Scheffrahn[2], Patrick J. Keeling[1,*]

[1] Department of Botany, University of British Columbia, Vancouver, British Columbia, Canada
[2] Fort Lauderdale Research & Education Center, University of Florida, Davie, Florida, USA

[*]Corresponding author; pkeeling@mail.ubc.ca

Supplementary Figure Legends

Supplementary Figure S1. Bayesian phylogeny of SSU rRNA sequences of the class Cristamonadea (1527 bp) including the sequence from *M. yanesha*. The tree is rooted with sequences from the closest known relatives to the Cristamonadea, members of the class Tritrichomonadea. Sequences obtained in this study are in bold type. Taxon names include GenBank accession numbers and the name of the termite host. Numerical values at nodes indicate Bayesian posterior probability (where greater than 0.90) and % ML bootstrap support (out of 1,000 replicates, where in agreement with the Bayesian topology and greater than 60%). Sequences assigned to or likely belonging to the joeniid genera *Joenia* and *Joenina* are denoted by a thick vertical bar. Tree figures were generated using the FigTree software v1.4.4 (http://tree.bio.ed.ac.uk/software/figtree/) and edited in Adobe Illustrator (https://adobe.com/products/illustrator).

Supplementary Figure S2. Bayesian phylogeny of SSU rRNA sequences of the class Cristamonadea (1526 bp) including the sequence from *Joenoides intermedia*. The tree is rooted with sequences from the closest known relatives to the Cristamonadea, members of the class Tritrichomonadea. Sequences obtained in this study are in bold type. Taxon names include GenBank accession numbers and the name of the termite host. Numerical values at nodes indicate Bayesian posterior probability (where greater than 0.90) and % ML bootstrap support (out of 1,000 replicates, where in agreement with the Bayesian topology and greater than 60%). Sequences assigned to or likely belonging to the joeniid genera *Joenia* and *Joenina* are denoted by a thick vertical bar. Tree figures were generated using the FigTree software v1.4.4 (http://tree.bio.ed.ac.uk/software/figtree/) and edited in Adobe Illustrator (https://adobe.com/products/illustrator).

Supplementary Video S1. Differential interference contrast (DIC) microscopy video clips of new *Devescovina*, *Macrotrichomonas*, and *Runanympha* specimens. Symbionts were observed with an Axioplan 2 DIC microscope (Zeiss) and multiple cells of each symbiont species were filmed with a 3CCD HD video camera XL H1S (Canon).

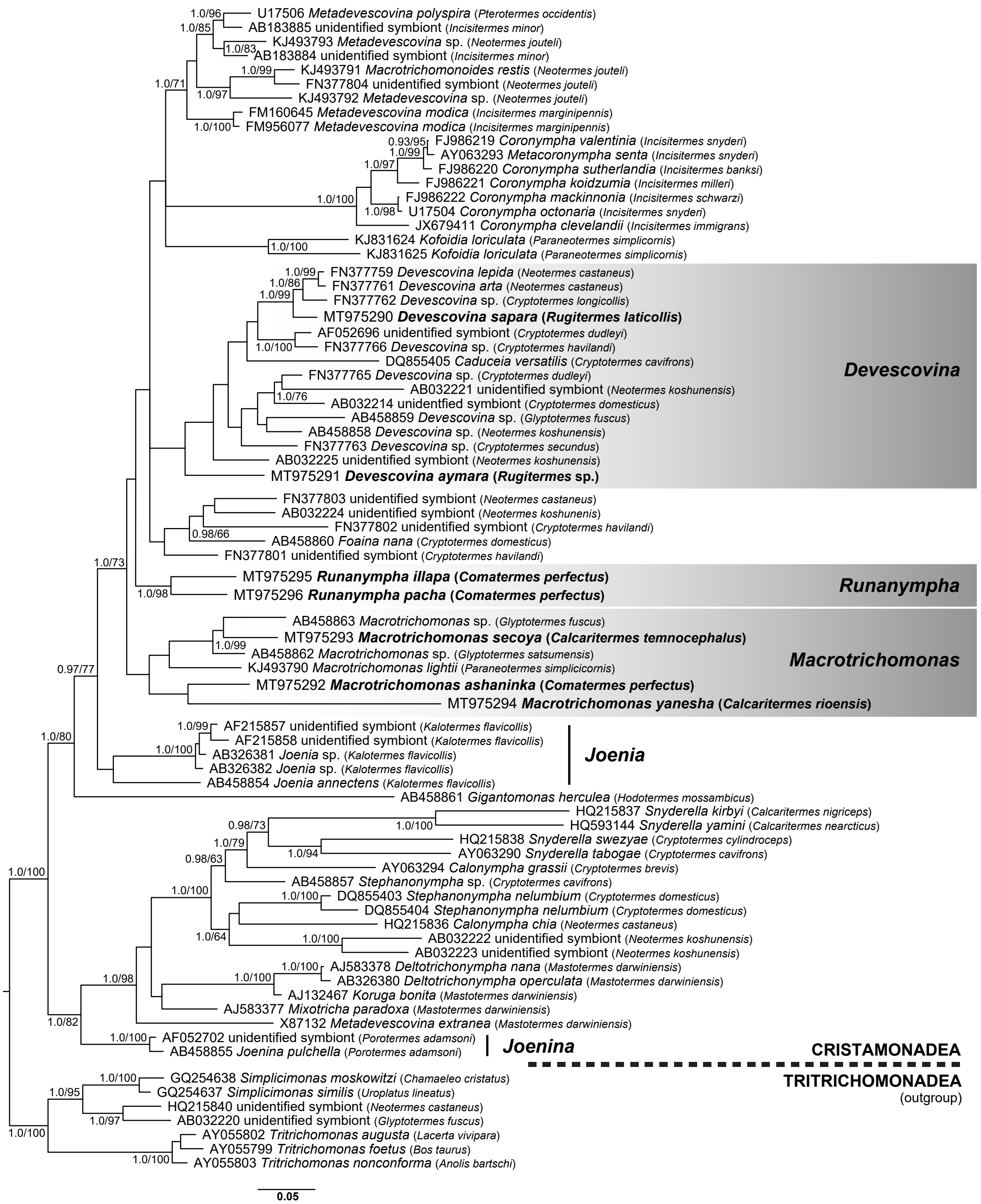

Supplementary Figure S1.

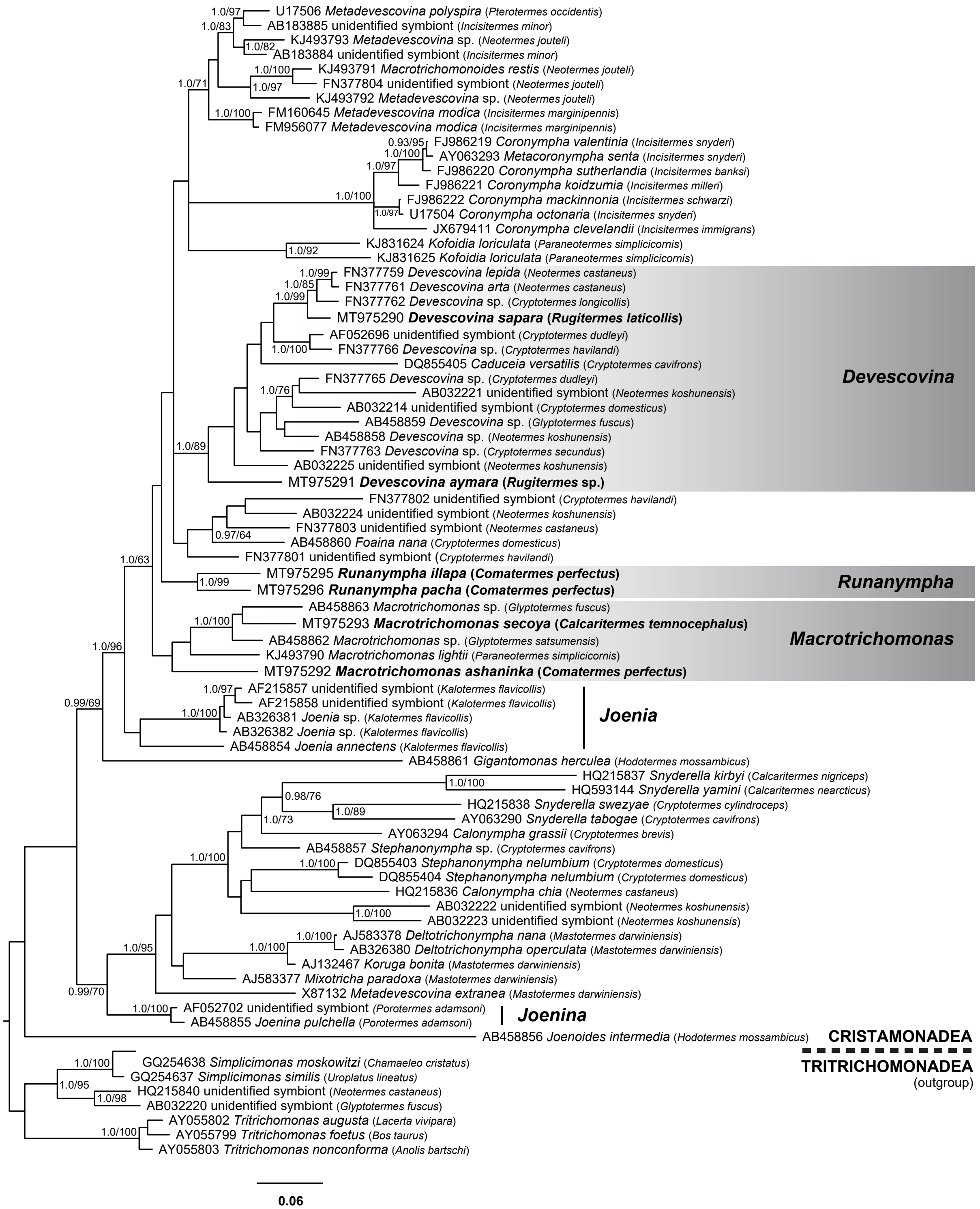

Supplementary Figure S2.

Supplementary Tables

Supplementary Table S1. Mean pairwise identities of SSU rRNA sequences within new Cristamonadea species and across species. Mean pairwise identity values across species are calculated between each new cristamonad specimen and the sequence with which the species shares the highest mean pairwise identity (also listed in the table). Sequences that were obtained in this study are marked with an asterisk (*) whereas sequences collected from Genbank are presented with Genbank accession numbers.

**Species**	**Mean pairwise distance of sequences within species**	**Mean pairwise distance to closest sequence in the tree**	**Closest sequence in the tree**
Devescovina sapara	99.21 %	97.43%	FN377761.1 – *Devescovina arta*
Devescovina aymara	98.51%	94.21%	FN377763.1 – *Devescovina* sp.
Macrotrichomonas ashaninka	97.62%	93.74%	AB458862.1 – *Macrotrichomonas* sp.
Macrotrichomonas secoya	98.12%	95.77%	AB458862.1 – *Macrotrichomonas* sp.
Macrotrichomonas yanesha	99.13%	89.96%	**Macrotrichomonas ashaninka* sequence 14_50_38Mac17_32
Runanympha illapa	97.46%	95.59%	**Runanympha pacha* sequence 19_10_43_17Placo_8
Runanympha pacha	96.82%	95.58%	**Runanympha illapa* sequence 18_10_26_35Joe_37_2
